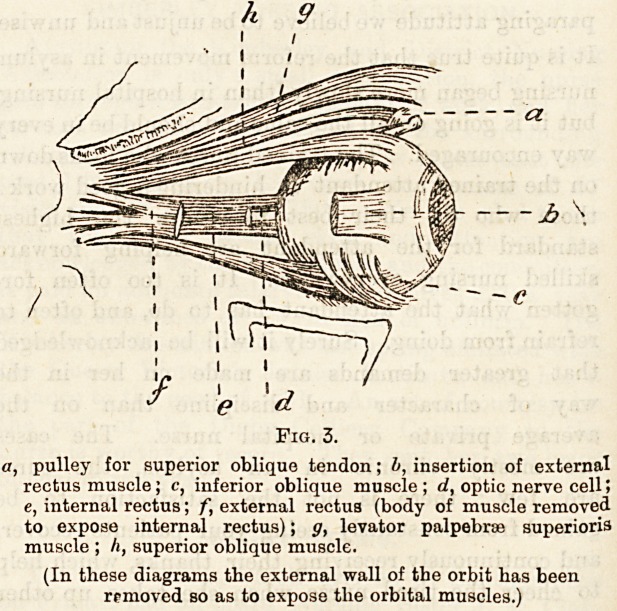# The Hospital. Nursing Section

**Published:** 1903-02-07

**Authors:** 


### The Hospital.

**Nursing Section.**

Contributions for this Section of "The Hospital" should be addressed to the Editor, "The Hospital" Nursing Section, 28 & 29 Southampton Street, Strand, London, W.C.

No. 854.—Vol. XXXIII. *SATURDAY, FEBRUARY 7, 1903.*

### Notes on News from the Nursing World.

### THE QUEEN'S VISIT TO WOOLWICH.

No date has at present been fixed for the visit of the Queen to the Herbert Hospital, Woolwich. The new quarters for the nurses are making steady progress, but they will not be finished for some little time. The new matron will be in residence when her Majesty inspects the Home.

### ROYALTY AND NURSING.

The younger members of the Royal Family are showing their practical interest in nursing. Princess Margaret of Connaught has delighted the supporters of the Bagshot Nursing Association by consenting to open a bazaar in aid of its funds next Wednesday. There is no doubt that the fact of the young Princess having undertaken to perform the function will ensure a good attendance on the occasion. This will be her first public appearance as a principal in a ceremony. The Hampstead Nursing Association must also be congratulated upon the promise of Princess Christian to be present at an afternoon concert to be held on March 21st at the Town Hall, Haverstock Hill, on behalf of the movement.

### QUEEN VICTORIA'S NURSE.

It will be remembered that during the last illness of Queen Victoria she was nursed by Miss M. A Soal, who, since April 1898, has resided on the Osborne estate for the purpose of ministering to the sick. She is now leaving the Isle of Wight for London, but how greatly her services have been appreciated by the residents on the Osborne estate is shown by the fact that they have just presented her with a handsome dressing-case as an expression of their regard and good wishes. Miss Soal, in addition to four years' training at the Royal Free Hospital, was a probationer for some time at the Seamen's Hospital, Greenwich; she has been a district nurse at Croydon and Wandsworth, and holds the certificate of the City of London Lying-in Hospital.

### AN ECHO OF THE SIEGE OF MAFEKING.

During the war in South Africa many nurses achieved distinction and none more than those who were engaged in tending the sick through the protracted siege of Mafeking. The services of these devoted women have not been disregarded, but we are glad to notice that Mr. Chamberlain in his eloquent reference to the siege did not overlook them. In his speech at the recreation ground he praised not only the skill and unfailing resource of the commander, the bravery of the volunteers and the town guard, but also "the pluck and humanity of the nursing sisters." This tribute of the Colonial Secretary will be as acceptable as it is just.

### THE ARCHBISHOP AND THE NURSES' HOME.

The Archbishop of Dublin has been preaching at St. Stephen's, Dublin, on behalf of St. Patrick's Nurses' Home. In the course of his sermon, his Grace alluded to the record of the home as "splendid," and mentioned that during the year the new cases attended by the nurses amounted to 2,100, the number of visits being nearly 40,000. He made a strong appeal to the congregation to contribute liberally to an institution which he said most thoroughly deserves their aid. The nurses attached to the home, whether they visit the sick poor in Dublin, or work as district nurses in different parts of Ireland, have the confidence of all classes, from the Archbishop and the Lord Lieutenant—who contributed £25 towards the home as a thank-offering for the recovery of Lady Dudley—to those who derive personal benefit from their ministry.

### NURSES CONVERSAZIONE AT ST. THOMAS'S HOSPITAL.

A very large number of nurses and others accepted the invitations to the nurses' annual entertainment at St. Thomas's Hospital on Friday last. The result was a "crush," and a most successful evening. The long corridor was decorated with Chinese lanterns, palms, and there was a lavish provision of amusements. In the Central Hall a succession of biograph pictures, Hospital Pierrots, and selections by Herr Wurm's Viennese White Band was kept up from eight o'clock till eleven, and some novel side-shows also attracted a good deal of attention. One of these was an exhibition of glass-blowing, where the process of making retorts and other blown-glass objects was shown, while in another corner one came upon a potter "thumping his wet clay," and surrounded by an admiring group of spectators intent on watching the rapid evolution of vases and jars. In a dark room off the corridor was an exhibition of Roentgen Rays; and scattered up and down were half-a-dozen palmists, each besieged by people anxious to be told their character or "fortune." The new casualty department, with its clean white-tiled walls, was also on view for those who cared to face the cool fresh air. The Hospital Pierrots gave two performances during the evening. The troupe consisted of Professor Aikin Payne and Sir Vical Vane (sopranos); Herr Salmonio Gutti and Lord Osis (altos); Herr Follicle and Prince Gargarisma of Borax (tenors); and Signor Sawboni, Mr Albino and Count R. Terry Forceps (basses). Their singing of "The Three Chafers," "Sweet and Low," and "O, Who will o'er the Downs so Free," was particularly good. Among the biograph pictures were her Majesty the Queen presenting medals to the Yeomanry hospital staff, and the Royal procession through the City on October 25th, with the ceremony at Temple Bar.

### A LIBEL ON LEEDS GENERAL INFIRMARY.

It was stated the other day in a Yorkshire daily paper that the Governors of the Leeds General Infirmary "will not appoint a nurse who is a Roman Catholic, no matter what her qualifications may be." Local journals should be well informed, but in this instance there has been a mistake. As a matter of fact, there is now a Roman Catholic nurse working on the staff of the Leeds General Infirmary, and we understand, on the best authority, that the Governors are most broad with regard to the religious question.

### NURSING BY ORDERLIES.

This week we publish some interesting comments by a member of the Royal Army Medical Corps in South Africa upon an article by a Reserve Sister on "Nursing by Orderlies" which appeared in our columns in November. The writer is attached to the General Hospital at Kroonstad, and went through the late war. We agree with him that it is a matter for regret that an orderly who shows an aptitude for nursing should not be allowed to remain in hospital instead of being drifted to other employment for which he is perhaps less suited. Of course we do not for one moment doubt that the writer in his own particular case was in the habit of doing all the nursing of the patients whilst the sisters chatted to the orderly officer, sewed, or read during the night, and "gallivanted" about with officers during the day, but we have reason to believe that the nurses whom he so scornfully calls "angels of mercy" have occasionally some more serious occupation than flirting and "gallivanting," and were now and then gratefully spoken of by patients whom they had tended with devotion. This, too, in spite of the fact that a sister is only a woman who "has always been recognised as the weaker vessel, which has to be guided by a man and not *vice versâ*." If the average orderly's feeling towards the sister of his ward be faithfully represented by our correspondent at Kroonstad, the existing system must be, indeed, deplorable.

### A DEFICIT COVERED BY A DANCE.

The Bradford District Nursing Association, which held their annual meeting last month, were rescued from an unpleasant position in the nick of time. The financial report for their second year showed a deficit of £73 17s., but thanks to a ball promoted on behalf of the organisation, and held on the eve of the meeting, they were placed in funds, the ball realising the handsome sum of £80. This enables the Association to start the year with a clean sheet, but they will be wise not to rely upon another ball to set them straight. The expenses, which amount to £950 a year, should be covered by subscriptions, and with such a record as 38,000 visits to the sick poor of Bradford, this should not be very difficult. If, then, exceptional help is offered, the proceeds will be available for the basis of the reserve which it should be the object of all nursing organisations to possess.

### THE ADVOCATES OF RETROGRESSION IN WORKHOUSE NURSING.

The *Poor Law Officers' Journal* is very angry with Mr. Sydney Holland because in a letter addressed to the editor of that paper he said, "If I were a betting man I would bet that the 'qualified' nurse is never launched into existence, despite the sneer of the *Poor Law Officers' Journal* at the cost of those who oppose this retrograde step"; but it is compelled to admit the possibility that Mr. Holland's prophetic insight may prove reliable. Its consolation is that even if this proves to be the case, "no more than a sort of Pyrrhic victory will have been achieved by the Workhouse Infirmary Nursing Association and the medical men joined with it." In plain English, our contemporary is of opinion that the proposal to create "qualified" nurses is conceived in the interests of the aged and infirm patients in the county workhouse infirmaries, and that if it be abandoned, these poor people will be the sufferers. But in what respect the aged and infirm patients can really gain from the substitution of a new order of "qualified" nurses for the assistant nurses of the present time, is not explained. The fact is that the inevitable result of this, or any other retrograde step, must be injurious to the patients. The elevation of the standard of nursing has always been urged in these columns, primarily on the ground that the more highly trained the nurses are the better it must be for the sick and suffering.

### THE RIGHT PRINCIPLE AT BUXTON.

It is rather surprising that the committee of the Buxton District Nursing Association should have to present a report showing a balance on the wrong side of £12. Buxton contains plenty of well-to-do persons who ought easily to find between £200 and £300, the sum needed to relieve the committee of all anxiety. We are glad to notice that the working classes do their share. The chairman in his speech at the annual meeting stated that there were now over 500 subscribers, and that the number indicated the considerable interest taken in the sick poor. He also pointed out that those who read the report carefully would find that the persons attended were "in all cases those who could not afford to pay for a nurse." This is the principle upon which such organisation should always be conducted.

### A NEW DEPARTURE AT TOTNES.

The Queen's nurse at Totnes has gained high encomiums, not only from the inhabitants of the town, but also from Miss Peter, who again reports that the working of the District Association has been very satisfactory. As to figures, 232 of the working classes paid a penny a week for the whole of last year, and the total amount received in weekly subscriptions was £53 18s. 5d. This is practical testimony of the work of the Association and appreciation of the nurse's services. The latter last year paid 3,710 visits to 168 patients. The total amount raised was £167 3s. 1d., and the committee have been able to place £15 to the reserve fund and carry forward £13 10s. 8d. The Association committee have now decided to establish a Samaritan Fund, the purpose of which is to give extra nourishing food to bread-winners in the last stages of their convalescence, and if this effort, which will be entirely dependent on voluntary contributions, is as well supported as the Nursing Fund, Totnes will have ample reason to be grateful to the Committee of Ladies for the benefits the Association has brought to it.

### A FLOURISHING ORGANISATION AT BARROW.

At the annual meeting of the Barrow District Nursing Association a highly satisfactory announcement was made. Messrs. Vickers, Sons and Maxim Limited, have increased their annual subscription from £25 to £100, in order to enable the Association to employ a nurse at Walney Island. The staff now, therefore, consists of four nurses, and so liberal were the donations from unusual sources last year that the credit balance is £459, a sufficient amount to meet the expenses of the present year. If this position can be maintained, Mrs. Wadham, the energetic vice-president of the organisation and its founder, will be able to feel that in five years she has achieved a most solid success. In these circumstances, the refusal of the Tram Company to allow the nurses to travel free, which was adversely commented upon at the meeting, is a matter of little moment. Instead of asking the Tram Company to grant the privilege of gratuitous rides to nurses, it would be far better to secure a contribution from the directors.

### A DEFICIENCY AT WALSALL.

At the annual meeting of the Walsall Victoria Nursing Institute the unsatisfactory announcement was made that there was a deficiency on the year's account of upwards of £80. This unfavourable state of affairs is all the more surprising in view of the fact that the labours of the district nurses were exceptionally heavy last year owing to an epidemic of typhoid fever. The figures showed that while in 1901 6,262 visits were paid and 308 cases nursed, the numbers in 1902 rose respectively to 7,994 and 434. The Walsall Board of Guardians recognise the work of the association by subscribing £25 per annum to the funds, but, as the chairman of the latter and other speakers urged, the sum is very small. It was compared to the contribution of the Leamington Guardians who have increased their subscription to the district nursing organisation from £70 to £170. The Walsall Guardians may plead that Leamington is a wealthier town, but the obvious rejoinder is that the proportion of sick poor in Walsall is far larger.

### FRICTION AT EXETER WORKHOUSE INFIRMARY

The Exeter Guardians have declined to comply with the request of the superintendent and assistant nurses at their workhouse that the superintendent might be allowed raw rations and cook them herself, and that the assistants might have their meat and vegetables cooked, as before, in the kitchen. The guardians have decided that the nurses' meat be cooked in the kitchen in one joint three times weekly, and that raw vegetables be served for the nurses' use, to be cooked in the hospital kitchen. The only exception with regard to rations is made for the night nurse; and as it would be impracticable for her to have her food at mid-day, the old practice of serving out raw rations is to be reverted to in her case. Although the medical officer recommended that the superintendent nurse's request should be acceded to, the guardians did not agree, and therefore, in consequence of having to attend to certain duties in the dinner hour, that official will have to endure at times the doubtful luxury of a cold or spoilt dinner. The assistant nurse has left, giving as her reason that it made her quite ill to wash and clean the expectoration cups; she has clearly mistaken her vocation.

### NURSES AND RE-VACCINATION.

Whatever latitude the law may allow to the private individual in respect to vaccination or re-vaccination, it is indispensable that persons who are employed in nursing the sick should submit to the judgment of experts. A nurse attached to the Ellesmere Workhouse Infirmary having stated that she "does not believe in re-vaccination," a member of the Board of Guardians attempted to justify her opinion, although there is small-pox in the neighbourhood. His plea for liberty did not, however, commend itself to his colleagues, and they very properly decided that the nurse should be required to undergo the operation at once. The alternative, we conclude, is her immediate dismissal. There is no room in public institutions for nurses who do not "believe" in re-vaccination.

### KIMBERLEY NURSING ASSOCIATION.

Although the Kimberley and District Nursing Association is only a small organisation, the nurse gets through a substantial amount of work, and her services are much appreciated in the surrounding villages. The report for last year states that she paid 1,984 visits to 126 cases, an increase of 57 visits and 18 cases over 1901. We are glad to see that the nurse, whose skill and attention are acknowledged by the medical men and the committee, is paid an adequate salary. She receives £80 a year and an allowance of £5 in lieu of uniform. The Kimberley Association was last spring affiliated with the Queen Victoria's Jubilee Institute, and has a balance to the good of £91. An act of courtesy on the part of the Digby Colliery Company to the nurse is worthy of record. They sent her two loads of coal in the year.

### SHORT ITEMS.

The s.s. *Orcana*, which arrived at Southampton on Friday, had on board Nursing Sister J. M. Clay, of the Army Nursing Service Reserve, who returns from South Africa in consequence of the reduction of establishment.—The offertory at the morning service in Westminster Abbey on Sunday, February 22nd, will be given to the Westminster District Nursing Association.—A course of six lectures on "Economics and Social Problems," arranged by the Committee on Social Education of the Charity Organisation Society, is being given by Mr. E. J. Urwick, M.A., at the Caxton Hall, Westminster, on consecutive Thursdays at 4.45 P.M.—At the invitation of the Rev. John Napleton, some of the students of Guy's Hospital gave a very successful entertainment on Wednesday evening last week to the inmates of the Hospital for poor French Protestants, Victoria Park Road, E.—On Thursday last week a comedy drama, entitled "The Quality of Mercy," was played for the entertainment of the in-patients of the Cancer Hospital, Fulham Road, by Miss Bessle and her friends.

### The Nursing Outlook.

"From magnanimity, all fear above;
From nobler recompense, above applause;
Which owes to man's short outlook all its charm."

### THE ASYLUM NURSE.

The terrible fire at Colney Hatch brings once more to the fore the fact that the asylum nurse needs to be of the highest character and thoroughly disciplined in self-control. There has always been a tendency to regard asylum nursing as on a lower level than hospital nursing, and not to demand of the attendant the same qualities required of the nurse. The feeling is evident even between the hospitals and asylums themselves; there is a constant bickering in nursing associations as to the status of the asylum nurse, and the Pension Fund is about the only great movement that has readily yielded her equality. This disparaging attitude we believe to be unjust and unwise. It is quite true that the reform movement in asylum nursing began more slowly than in hospital nursing, but it is going on all the same and should be in every way encouraged. The trained nurse who looks down on the trained attendant is hindering a good work; those who do their best to secure the highest standard for the attendant are helping forward skilled nursing everywhere. It is too often forgotten what the attendant has to do, and often to refrain from doing. Surely it will be acknowledged that greater demands are made on her in the way of character and discipline than on the average private or hospital nurse. The cases are mostly chronic in an asylum, the cures are few; there is not the satisfaction to be gained from constantly seeing your patients recover, and continuously receiving their thanks, which help to cheer the tired nurse when she takes up other cases. Again, the mentally afflicted are not only difficult to deal with, but they do not appeal to our pity in the same way that the physically afflicted do. Thus patients in asylums include for the most part patients with the vacant stare, the restless rolling eyes, the crouched huddled form of the melancholiac, the ceaseless, senseless babble of the childish adult, the wild and noisy maniac. Such patients are apt to cause a shudder and a certain repulsion in the average untrained person who would probably be spontaneously sympathetic to the poor sufferers in a hospital. Who can exaggerate the trials of a conscientious attendant in charge of senile and imbecile patients with their dirty habits and still more offensive tricks? Such patients are not responsible for this disgusting conduct, and the trained attendant knows and understands this, which enables her to quietly and conscientiously discharge the disagreeable duties demanded of her. We have seen on more than one occasion a lunatic spit on a nurse; or a nurse have a handful of hair torn out; and through it all the nurse has to maintain complete calm and self-control or the whole ward would soon be in an uproar.

Those are the every day things; and then there are also the moments, such as the fire at Colney Hatch last week, when we expect devotion to duty even unto death, if necessary; when we require the nurse to rescue her charges at the risk of her own life. And it is not only in cases of fire, but in cases which must now and again arise in asylums, of a patient regarded as harmless getting hold of some weapon, of a patient escaping to some desperate position, of two patients attacking one another. These cases are rare, but it is evident that when they do happen it is the duty of the nurse to fearlessly but deliberately interfere, unmindful of what the result may be to her personally. Really very few accidents happen to the nurses in these cases, merely because from long training they have moral and mental control over the patients. But the danger is there. A more subtle, a more common danger is the mental ill-health which often breaks down an attendant from too close companionship with the insane.

These facts are sufficient to demonstrate the right of the asylum nurse to a place on the highest plane. Remember also that she is nowadays subject to her three years' training, her examinations and her certificates, in the same way as the hospital nurse. Indeed, the Retreat at York has just instituted a fourth year of training in order to fit its nurses specially for going out to private cases. Owing to the narrow and mechanical routine of training in some of our hospitals, and the fact that they will train the nurses to suit the needs of the hospital, rather than the needs of the public, the ordinary nurse if she is sent to a mental case, generally makes a terrible failure. But, on the other hand, when an asylum nurse has been called in to a private mental case, the asylum methods have been too rough for the relations to stand. Therefore, this special training of mental nurses for private work is a great step forward. A difficult subject to touch is the fact that though many asylum nurses are thoroughly cultivated women, this cannot be said of them all. Because one or two women of title have tried three months in hospital, the hospital nurse can sometimes be rather a snob in these matters. Let us here, speaking as we know we do, for the vast majority of trained nurses, say that devotion to duty is far more important than blue blood, and that a self-disciplined character is a far higher proof of a gentlewoman than is a knowledge of Shakespeare. And so let the asylum nurse attain to the highest position, and let it be her pride to demand of herself that she fail not to hold it honourably.

### Lectures on Ophthalmic Nursing.

By A. S. Cobbledick, M.D., B.S.Lond., Senior Clinical Assistant Royal Eye Hospital, late House-Surgeon and Registrar, Royal Eye Hospital.

### LECTURE III.—ANATOMY OF EYEBALL (*Continued*).—THE EXTRINSIC OCULAR MUSCLES.

The retina extends forwards as far as the ciliary region, where its nervous elements disappear; it is, however, continued forward as a fine membrane to the edge of the pupil. The retina is supplied with blood by the arteria retinæ centralis, which enters the eyeball at the optic disc; it at once divides into two, an ascending and a descending branch, each of which again divides into an inner or nasal, and an outer or temporal branch; the blood is collected by two veins which do not unite but pass into the optic nerve as two small trunks.

The blood supply of the remainder of the eyeball is derived from the ciliary branches of the ophthalmic artery.

*The refractive media.*—The aqueous humour fills the anterior and posterior chambers of the eyeball, *i.e.* all that space which lies in front of the crystalline lens. The iris, in close contact with the lens, divides the space into two, but the fluid is free to pass from the posterior to the anterior chamber. It is important to remember that through the angle formed by the iris and cornea, the aqueous has access to some small lymphatic spaces, called the spaces of Fontana, which in turn are continuous with the canal of Schlemm.

Fig. 2.

*a*, Frontal sinus; *b*, levator palpebræ superioris; *c*, pulley of superior oblique tendon; *d*, tendon of superior oblique; *e*, external rectus muscle; *f*, superior rectus muscle; *g*, optic nerve; *h*, antium of Highmore; *k*, inferior rectus muscle; *l*, inferior oblique muscle.

*The crystalline lens*, as its name implies, is a transparent lens: it is bi-convex and its circumference is rounded. The convexity of the posterior surface of the lens is greater than the anterior. It keeps its position through being invested in a transparent capsule, which is fixed at its circumference by a suspensory ligament; this ligament is firmly fixed to the ciliary processes, and if traced backwards is found to be continuous with the membrane which encloses the vitreous. The fibres of which the lens is composed are concentrically arranged, not unlike the layers of which an onion is composed.

*The vitreous humour* is a clear fluid, somewhat thicker than the aqueous. It fills up the posterior portion of the eyeball: it is in contact with the lens and suspensory ligament anteriorly, but elsewhere it is in close contact with the retina, only separated from it by a thin membrane called the hyaloid membrane.

### The Extrinsic Muscles of the Eyeballs.

These are six in number, viz., four recti muscles and two oblique.

The recti are named from their position in relation to the eyeball, viz., internal, external, superior and inferior. The oblique muscles are superior and inferior.

All these muscles with the exception of the inferior oblique have their point of origin or fixation at the apex of the conical orbital cavity, in a circular tendinous band which encircles the optic foramen and crosses the sphenoidal fissure. As the muscles pass forwards they diverge from each other so as to take up their position in relation to the eyeball. All the muscles are long thin strips, which become flattened as they come in contact with the eyeball; at their point of insertion into the sclerotic, about a quarter of an inch behind the margin of the cornea, the muscles form a broad, thin membranous tendon.

Fig. 3.

*a*, pulley for superior oblique tendon; *b*, insertion of external rectus muscle; *c*, inferior oblique muscle; *d*, optic nerve cell; *e*, internal rectus; *f*, external rectus (body of muscle removed to expose internal rectus); *g*, levator palpebræ superioris muscle; *h*, superior oblique muscle.

(In these diagrams the external wall of the orbit has been removed so as to expose the orbital muscles.)

The superior and inferior *obliques* must be considered separately.

The superior oblique takes origin immediately above and to the inner side of the optic foramen, passes forwards close to the inner orbital wall and above the internal rectus muscle. As it reaches the forepart of the orbit it forms a thin rounded tendon which passes over a small pulley. At the pulley the direction of the tendon changes, passing backwards and outwards, beneath the superior rectus. At its insertion immediately beyond the external border of the superior rectus it forms a thin, broad membranous tendon which is inserted into the sclerotic.

The inferior oblique muscle is shorter and broader than those which have been considered. Its fixed point is on the floor of the orbit, at its anterior and internal portion, and is in close contact with the opening for the nasal duct. It passes outwards and slightly backwards below the inferior rectus to be inserted into the eyeball in its outer and posterior portion, nearer the optic nerve than the insertion of the superior oblique.

Whilst speaking of these muscles of the eyeball, it is necessary to allude to another orbital muscle, viz., the levator palpebræ superioris (the elevator of the upper eyelid). This muscle arises in the back of the orbit, above the optic foramen. It passes forwards immediately above the superior rectus, but is inserted into the fibrous portion of the upper lid. At its insertion it becomes tendinous and spread out in a fanlike manner.

All these extrinsic muscles—with the exception of two—are supplied by branches of the same nerve, viz., the third cranial or oculo-motor nerve. The exceptions are the external rectus, which is supplied by the sixth cranial nerve, and the superior oblique which receives its supply from the fourth cranial nerve.

### After Fifty: Or Poor Law Nurses and Pensions.

By Miss WILSON, Treasurer of the Workhouse Infirmary Nursing Association; Member of the Midwives' Board.

THE question of an adequate provision for Poor Law nurses in old age is so important that I am glad to avail myself of the Editor's invitation to place the facts of the situation before the readers of THE HOSPITAL. The problem must appeal to all who are interested in the nursing profession, and my own attention and sympathy have been so often drawn to it in a very sad and urgent manner that I am enabled to write with some experience.

I do not know that the average life of a nurse is unusually short, but her average length of healthy life is undoubtedly shorter than that of the ordinary worker. When, as is often the case, body, brain and nerves are kept constantly at their full stretch, the price must necessarily be paid. If this be admitted, the question is, are nurses—and I deal only with Poor Law nurses—now a great and increasing army of nearly 5,470 prepared to face this fact in a spirit of wisdom and foresight?

### THE DISTRIBUTION OF FIGURES.

After a somewhat exhaustive inquiry, in which I have been most kindly helped by many of the Poor Law matrons of infirmaries, I find that out of a total of 807 nurses, employed in 20 Metropolitan infirmaries, 108 are making provision for the future through the agency of the Superannuation Act, and 54 through the Royal National Pension Fund. These figures do not include probationers. In four large provincial infirmaries, in which the total nursing staff, including probationers, amounts to 360, 15 nurses are making provision through the Superannuation Act, and 20 through the Pension Fund.

The distribution of the figures is extremely interesting. For instance, at one infirmary only two nurses belong to the Royal National Pension Fund for Nurses, while 15 have joined the Poor Law Officers' Superannuation Fund. Whereas at another large infirmary the number belonging to the Royal National Pension Fund is eight, and to the Superannuation Fund three.

In considering these inadequate figures we may still hope that there may be some nurses who, while not contributing to either of these funds, are yet making provision through the agencies of the General Post Office, Building Societies, or through the Prudential Society—the last, it may be noted, has the convenient practice of directly collecting premiums. But a fairly wide knowledge of Poor Law nurses leads me to think that this is not very generally the case, and I fear that, roughly speaking, we must consider the figures given as fairly representative of the present saving capacity of a very large and growing body of women workers.

### DETERRENTS TO SAVING.

The causes which act as a deterrent to saving in the case of women are obvious; they have only lately become workers in any wide or independent sense of the word, and their responsibilities still sit lightly on them. Many also feel responsible only for themselves. Because they have not been obliged to save for a family, as men are expected to do, they think that money earned may fairly be spent, and that their future will adjust itself in some agreeable but highly indefinite way. Either the duty of self-support, when it becomes a burden, they hope will be removed by stronger hands, or later on they propose to take up private nursing and then begin to "save." In any case the matter is not very seriously considered by the majority, and it must, I think, be concluded from my figures that the future does not weigh heavily on the rank and file of our nurses. That this is the case in Poor Law work shows either an extraordinary power of hope or an incapacity to realise facts as they are; for to my mind one of the saddest experiences brought daily before the infirmary nurse is the way in which, by a gradual loss in means or savings or by ill-health, the well-to-do and highly respectable are brought—often most unwillingly—to be inmates of our State-aided institutions. One would think that these sad instances would at any rate preach a sermon of thrift, but it falls on deaf ears. The sublime confidence that such calamities "cannot possibly happen in my case" doubtless helps a willing blindness to the future. But experience has another duty beyond a mere smile and shake of the head.

### THE OPINIONS OF MATRONS.

I append extracts from letters from the matrons of a few large infirmaries, which give in their view some of the reasons for the present state of things.

The matron of an important infirmary and training school writes: "My staff consists of over a hundred nurses, and the number of nurses in the Pension Fund is 10, and under the Superannuation Act three. As one of this number is in both funds, the number who are making any provision for old age is only 12, I don't see how nurses can make much; they have to pay such a greal deal for so very inadequate an annuity. Can you expect a young nurse to forego, as she must, all pleasures to secure £15 a year at 50?"

Another matron says: "My own experience is that nurses, as a class, are not willing to belong to anything for which they have to pay, and I think that the retiring age for nurses under the Poor Law should be 50 or 55 instead of 60 or 65."

The matron of an infirmary in which there are a large number of probationers, writes: "Probationers' salaries are so small I really don't see how they can afford to save out of £10 and £15 a year unless they have friends who are able to help. Often I fear it is the reverse of being helped."

Another matron writes: "I regret that more do not belong to the Pension Fund, but the average nurse's salary is small. . . . There is, I suppose, perhaps 1 (nurse or servant) in 100 who stays in the Poor Law service until she is old enough to reap any benefit from the Fund, and the small annual subscription drawn from the many subordinate officials forced to join goes to swell the pension paid to the higher officials who have found it worth while to hold office for a sufficient number of years under the Poor Law."

### AFTER FIFTY: OR POOR LAW NURSES AND PENSIONS—*Continued.*

The matron of a London infirmary writes: "The reason I think so few have joined the Royal National Pension Fund is that the premiums are high, and the nurses prefer to wait until their salaries increase sufficiently after completing three years' training. It is also exceedingly difficult to persuade young people to begin early to make provision for the future. Almost without exception the nurses contract out of the Superannuation Fund. Had it been possible to alter the age limit when the amendment was passed, I think it would have made a difference, and perhaps been helpful to the nurses. As it is, I find (in the working of the Poor Law Officers' Superannuation Act) my opinion is only more confirmed of the many difficulties it creates in the working of an institution and keeping the staff efficient."

The matron of a large London infirmary writes: "I think that the nurses are very unfairly placed in reference to the Poor Law Officers' Superannuation, the age limit when they can commence training, rendering it almost impossible for them to benefit in comparison with masters, stewards, clerks, many of whom begin their work as lads."

### Arguments against Thrift.

These statements, with others I constantly hear, fall under two classes of argument:—(*a*) Young nurses cannot save because they cannot be expected to forego the pleasures natural to their age. (*b*) Nurses cannot save because neither the Pension Fund nor the Superannuation Act is really available for their needs. With the first argument, except as regards probationers, I cannot say I am in entire accord, and even probationers could, in some cases, enter for a policy, and when a woman has finished her training, I think, unless her circumstances are very peculiar, she should regard some form of provision for illness or age as a moral duty. She is at that period, as a rule, in good health, and should expect to receive an annual increase of salary. She can then join a pension fund at a much lower premium than later in life, and she has none of the drawbacks which may cripple her and act as a handicap to her added experience later on; defective eyesight and hearing, the effects of rheumatic or typhoid fever, may stand in the way of obtaining a really good salary. The later half of life Bishop Warburton truly characterised as often "a losing game," and if a nurse waits till she has entered on that part of life, her difficulties in saving will be enormously increased. To form economical habits in early life, some self-denial will certainly be required, but not, I think, of such a serious kind that it will sour a character which has already had some discipline; and to nurses in these days, pleasures are often kindly provided by appreciative friends in the form of theatre and concert tickets, etc., which were almost unheard of twenty years ago.

### Family Claims.

The claims of family are, I admit, a serious offset against saving, and the way in which these claims are sometimes pressed is notoriously selfish. Why it should be thought that a woman owes no duties to her own future welfare as a man does to his is, to say the least, puzzling. After full consideration I am convinced that nurses are more frequently the supporters of the family scapegrace than almost any other members of the community. Nurses, too, are often told by their family that they "have a salary, and no expenses," and a general agreement appears to exist that there is a certain amount of danger for a woman in so enviable a position, and that the family must take immediate and effective steps in order to minimise the risk. It is often difficult to fight against this attitude, and strong natural affection is of course inclined to give freely. Neither side seems to realise the serious dangers to a nurse's health inherent in her profession, by which any day the willing giver may become the unwilling burden. When there is, as too often, a clear and legitimate family duty, such as the support of a mother, or of an invalid sister, the manner in which nurses fulfil it is indeed admirable. I know at the moment of a nurse who, after being operated on for cancer three times, is now by her work helping to maintain an aged father and an invalid husband, both of whom but for her exertions would probably be at the mercy of the world.

Another case of what has been done in self-help may be interesting. A nurse I knew was so much impressed by the sad facts of the long illness and death of a friendless nurse in middle life, in the sick ward of the same rural workhouse in which she also worked, that, although elderly, she without delay applied to the Pension Fund for a policy and for some time paid £20 out of the salary of £25, in order as she said, "that she might live her later life independently." She was without relations and felt a great effort of self-denial was needful in order to make up for her previous lack of foresight.

Such heroism as the first case mentioned, and such wisdom as is shown in the second are beyond praise; but we must be thankful that the need for these qualities in a high degree is exceptional. A nurse above all women should be the first to keep before her eyes the fact that health is uncertain, dependent age most pitiable, and that her simple duty is to live and to be of use. Marriage, the bourne of some eyes, is not the end of all responsibility. A fair proportion of married nurses, even if they do not become widows, are still often obliged to return to their work. Indeed, my observation leads me to the conclusion that either nurses are bad judges of character in husbands, or else that idle men have a particularly clear idea of the advantages involved in marrying a woman who is equipped with a calling which will provide for two!

(*To be Continued.*)

### State Registration of Nurses: A Rejoinder.

By HELEN TODD, Matron of the National Sanatorium for Consumption, Bournemouth.

"A Hospital Sister" draws attention to what she considers some of the difficulties in the path of "State Registration." I trust I may be allowed to try and show her that the very details which she finds stumbling blocks are really all striking arguments in favour of the scheme. Both the friends and opponents of State Registration undoubtedly agree with "A Hospital Sister" in deploring the manner in which the title "nurse" is too often used as a cloak for the vicious, the baby-farmer, and the criminal; but whereas the promoters of registration have purposed to band themselves together in order that they may try to devise some means of protection for the fair name of their beloved profession, the opponents are contented with sitting still and crying "Alack, my sister!"

### Why Registration is Sought.

"A Hospital Sister" does not appear to quite understand why we are working for registration. She acknowledges that the Midwives Act was passed to protect the poor "from the danger of being attended by unqualified and ignorant persons;" she has evidently not grasped the fact that it is for this very reason that registration is being sought by trained nurses. We recognise sadly that times are changed since nursing became a profession for educated women; then patients, and an orderly is responsible for the neatness and cleanliness of a ward, drawing and issuing meals, stores, etc. But a sister gradually puts the nursing bit by bit on to the orderly's shoulders, until at the finish she does nothing at all except taking temperatures and distributing medicines, which leaves her the rest of the day free to play tennis and gallivant about with officers, a thing which is never neglected whenever an opportunity offers. I think the reasons why so many civil nurses joined the Army Reserve and came to the war can be summed up in a very few words—a woman's privilege, curiosity, more latitude, better pay, less work, and a desire to escape the drudgery of civil hospitals. These are the ladies the misguided public send to the war to nurse the sick and wounded, and call them "Angels of Mercy." Your correspondent is quite right in saying that a Reserve sister's position is an impossible one, for the simple reason that they do not use the one great thing that is necessary, and that is tact. Men will not submit to be controlled by women. It is only natural, for a woman has always been recognised as the weaker vessel, which has to be guided by a man and not *vice versâ*. When staff nurses are introduced into the R.A.M.C. it will do away with the necessity for ward-masters, and will be the signal for the dissolution of the corps, as the orderlies would then become lackeys. Giving sisters a commission is out of the question altogether, as they would expect to be saluted as an officer, and the men would never submit to that. In all branches of commerce women have tried their hardest to usurp men's places. But there is no doubt in this case that it will be an utter failure.

### Novelties for Nurses.

BY OUR SHOPPING CORRESPONDENT.

WATERPROOF GARMENTS AND GOODS.

MESSRS. ANDERSON AND ANDERSON, of 37 Queen Victoria Street, are holding a sale of their wares, commencing on February 11th. The quality of this firm's manufactures is so excellent that nurses should take advantage of the reductions offered to acquire some of the most useful articles advertised in the catalogue issued. The waterproof capes will prove most serviceable to nurses who bicycle, whilst the various appliances for the sick-room, such as water-beds, pillows, and bottles are reduced to prices which are lower than those asked for second-rate articles elsewhere. Messrs. Anderson's hot-water bottles deserve special notice, as they are quite unsurpassable in durability—a feature that should give them a monopoly in this direction when once tested by use. There is no worse purchase than bad india-rubber goods, and from this those who purchase from Messrs. Anderson and Anderson are protected.

### Wants and Workers.

TO SOUTH AFRICAN READERS.

SISTER BUCHER (late A.N.S.), Box 116, Kimberley, will gladly forward THE HOSPITAL current monthly number to any South African address.

### Death in Our Ranks.

A CORRESPONDENT WRITES: On January 21st, Nurse E. Hyde, a probationer at the Nottingham Infirmary, Bagthorpe, died after a long and very trying illness. Her gentle, amiable disposition, together with the patience and fortitude with which she bore her illness, endeared her to all who knew her; and her promising career cut short, at such an early age, has caused the deepest regret to her many friends. The funeral took place at the little village of Woodville, near Burton-on-Trent, where a large number of the staff journeyed from Nottingham to show their esteem for their late comrade, and placed a beautiful floral tribute on the grave.

### Appointments.

[No charge is made for announcements under this head, and we are always glad to receive, and publish, appointments. But it is essential that in all cases the school of training should be given.]

FRIEDENHEIM HOSPITAL, LONDON, N.W.—Miss Ethel Cox has been appointed charge nurse. She was trained at the Royal Albert Hospital, Devonport.

ILKLEY HOSPITAL AND CONVALESCENT HOME.—Miss Frances Leng has been appointed matron. She was trained at Royal South Hants Hospital, Southampton, been sister at Richmond and Truro, and matron at Truro for nearly two years during the matron's absence in South Africa.

KETTERING INFECTIOUS HOSPITAL.—Miss E. Weis has been appointed matron. She was trained at the City of Glasgow Fever Hospital, and has since been nurse at Darlington General Hospital, and sister at Bolton Borough Hospital. She has also done private nursing.

MACCLESFIELD GENERAL INFIRMARY.—Miss Annie W. McCloskey has been appointed sister of the women's wards. She was trained at the Sheffield Royal Hospital, has done private nursing in London and Glasgow, and for the last 14 months has been sister at the Sheffield Royal Hospital.

NATIONAL HOSPITAL FOR CONSUMPTION, NEWCASTLE, CO. WICKLOW.—Miss J. Collier has been appointed sister. She was trained at the Norfolk and Norwich Hospital, Norwich, subsequently becoming a member of the Birmingham District Nursing Society.

PARKFIELD NURSING HOME, LIVERPOOL.—Miss Eva Jones has been appointed sister. She was trained at Mill Road Infirmary and Ladies Charity Hospital, Liverpool, where she was sister for two years. She was on the nursing staff of the Nightingale Home, Southport, for 18 months, aud has also done private nursing on her own account. She holds the L.O.S. certificate.

PONTYPRIDD DISTRICT NURSING ASSOCIATION.—Miss Lucy Hill has been appointed superintendent nurse. She was trained at Crumpsall Infirmary, Manchester, and the Workhouse Infirmary, Newcastle-on-Tyne. She has since been connected for nearly two years with a surgical home in Manchester, has had training in district work in Salford, and has worked as Queen's Nurse at Darwen and Merthyr Vale.

THE MEATH HOME FOR EPILEPTICS, GODALMING.—Miss Isabel Lawrence has been appointed lady superintendent. She was trained at Crumpsall Infirmary, Manchester, held the post of charge nurse at the Cancer Hospital, Brompton, for four years, and for the last five years has been sister at the National Hospital, Queen Square.

### TRAVEL NOTES AND QUERIES.

SWITZERLAND IN SEPTEMBER (Nurse H.).—At Wengen, just under the Wengern Alp, you could get accommodation at 5 francs per day, a beautiful situation not very far from Lauterbrunnen. Then there is Meirengen, close to the Brunig Pass, Lake of Brienz, etc., not far from Interlaken and Thun. A lovely spot; terms about the same. Or in quite a different direction, there are Lausanne, Vevey, Montreux, etc. . . . on the Lake of Geneva. The cost may be arranged on much the same terms at all these places. I do not think that what you call homely pensions can be often met with now, though many of them are extremely comfortable. At almost all French is the language spoken generally. You cannot compare the Lakes of Geneva and Lucerne; their style of scenery is very different, but both are, in my opinion, equally fine. Tourist circular tickets can be had to either. When you have settled a little more where you wish to go and nearer the time write to me again and I will give you addresses. In the interim, if I were you, I would read some books on Switzerland, such as Whymper's; it adds greatly to one's enjoyment.

### Echoes from the Outside World.

### Movements of Royalty.

THE King drove to Buckingham Palace from Windsor on Saturday morning in his motor car and returned later by railroad. The same train contained several ladies and gentlemen who had received invitations to the Castle, including Mr. H. White (the American Chargé d'Affaires) and Mrs. White and the Bishop of London. In the evening, by command of the King, Mr. Sousa's American band played before the King, the Queen, and their guests in the Waterloo Chamber. The band consisted of 60 performers, who had travelled by a special train from Manchester in the afternoon, where they had abandoned the two performances advertised so as to obey the Royal behest. Halfway through the programme the King requested Mr. Sousa to play some of his own American compositions, and amongst other pieces "Washington Post," "Hands across the Sea," and "The Stars and Stripes" were performed. Subsequently the King, who seemed much pleased with the entertainment, shook hands with Mr. Sousa, and Mrs. Sousa was presented to their Majesties.

### Indisposition of the King.

ON Sunday, the King and Queen and other members of the Royal Family attended Divine Service in the private Chapel at Windsor Castle. On Monday, between 11 and 12, his Majesty, accompanied by the Prince of Wales and Prince Edward of Wales, drove in a close carriage to the Home Park near the Datchet Road. Here preparations had been made for the planting of three young elms, as the beginning of an avenue of a hundred to be placed in position later. The Mayor of Windsor met the Royal party, and the King, using a silver ebony-handled spade, planted the first tree. The Prince of Wales followed, and then his little son, who found the large spade rather difficult to wield, performed the task. In doing so he scattered some of the earth over the Mayor's feet. The King exclaimed, "Eddie, you are trying to plant the Mayor!" His Majesty was thought to be looking remarkably well, but soon after his return home it transpired that Sir Francis Laking had visited the Castle, and, as he found that the King had a feverish cold, he advised that the journey to Chatsworth, which was to have been undertaken at two o'clock, should be postponed. On Tuesday it transpired that his Majesty was suffering from a mild attack of influenza; and in consequence, with much regret, it was decided to abandon for the present the Royal visit to the Duke and Duchess of Devonshire. The latest reports are that the progress of the King is quite satisfactory.

### The Disappointment in Derbyshire.

INTENSE disappointment was caused, not only in the Chatsworth district, but throughout Derbyshire, on Tuesday, when it was known that the Royal visit had been abandoned. Thousands of people had assembled on Monday, and again on Tuesday, in the neighbourhood of Chatsworth, or were journeying thither, in the hope of joining in the welcome to the King and Queen, and all hotel and other accommodation in the towns and villages for miles round had been engaged for the whole of the present week. Everyone, however, is hoping that the pleasure upon which they had counted has only been deferred for a short time.

### Mr. Chamberlain in Cape Colony.

DURING his stay at Mafeking, Mr. Chamberlain received Khama and other Protestant chiefs, and visited the Baralong *Stad.* Afterwards, in the course of a speech in the recreation ground, he said that the late war was a white man's war, and continued, "For reasons of policy, it was considered undesirable that men other than Europeans should take part in it. Had it been otherwise, the great Empire of India could have poured in tens of thousands of valiant, stalwart troops; there is not a colony, from the smallest to the greatest, over which I have the honour to preside, which would not also have poured in troops to show that it was prepared to share in the sacrifices as well as in the privileges of the Empire. The link which binds us appears to be as thin as gossamer, but let an enemy try to break through, and he will find it as strong as tempered steel." At Kimberley, where he had a splendid reception, he made two important speeches, the first in reply to an address of welcome, and a presentation to Mrs. Chamberlain of a silver bonbonnier containing five magnificent rough diamonds. In the former speech the Colonial Secretary said "each stone of the future Empire has been cemented by blood," a sentence likely to become historic; and in the latter, he eulogised Kimberley as "the mother of South Africa," and spoke of the late Cecil Rhodes in high terms. On Saturday Mr. Chamberlain arrived at Paardeberg, the encampment being pitched near the Modder, and close to where Cronje made his laager in the river bed; and on Tuesday he reached Bloemfontein. Both he and Mrs. Chamberlain were received with immense enthusiasm on the border of the Orange River town.

### The "Grey Ladies" at Blackheath.

ON Monday the tenth anniversary of the "Grey Ladies" was observed, and the Bishop of Southwark—whose sister, Miss Yeatman, was the first head of the body—admitted several new members, and delivered an address. The first house was dedicated by the new Archbishop of Canterbury, and there are now five houses with upwards of 40 members, who are unostentatiously doing splendid work among the poor in South London. The "Grey Ladies" belong to no particular school of thought in the Church, and are employed in parishes which include the clergy of all schools. They take no fees, are self-supporting, and give their services to the diocese.

### Gardiner and the Baby Farmers.

ON Friday William Gardiner, who was twice tried for his life in respect of the murder of Rose Harsent at Peasenhall, was released at the instance of the Crown, who entered a *nolle prosequi* in the case. The term *nolle prosequi* means "to be unwilling to prosecute," and is a familiar method, in civil procedure, of abandoning an action which has been commenced. Gardiner had been in prison 239 days on the date of his liberation. The Home Secretary declined to intervene in the case of Mrs. Sach and Mrs. Walters, the baby farmers condemned to death for murder, and they were accordingly executed on Tuesday morning.

### "Hiawatha" at the Albert Hall.

A VERY large audience assembled at the Albert Hall on Thursday last to listen to the "Song of Hiawatha," and by the enthusiasm of their plaudits evidently thoroughly appreciated the excellent manner in which the Trilogy was rendered. The Albert Hall choir have never sung better than in the quaint and fascinating choruses which describe the merriment of the marriage feast, as well as in the sad and impressive music which relates the death of Minnehaha and the grief of her stricken husband. The conductor was Sir Frederick Bridge, and the soloists were Madame Sobrino, Mr. Ben Davies, and Mr. Watkin Mills. The increasing favour in which Mr. Coleridge-Taylor's masterpiece is held was evidenced by the crowded state of the moderately-priced seats of the hall, and apparently the day is not far distant when the work of the young Anglo-Indian composer will be included amongst well-known classics. At the next concert of the society Sullivan's "Light of the World" will be given.

### A Book and its Story.

### A CHRONICLE OF THE REIGN OF ENGLAND'S LAST STUART SOVEREIGN.*

MR. JUSTIN MCCARTHY'S "Reign of Queen Anne" is an interesting survey of the period. No romantic associations cling to the memory of good Queen Anne, and yet in her personal history there were circumstances which should excite sympathy. Her married life was the reverse of happy. Prince George of Denmark, her consort, was "as characteristic a specimen of the good-for-nothing as any age or condition could have produced," and of her large family of children none survived infancy except the little Duke of Gloucester, who died in early childhood. "Anne came to the throne at a period which was illustrious in war, in politics, in literature, and art." It was an age which became a turning point, not only in the history of England, but in the history of Europe. It was then that modern politics took the place of the old political traditions, and the reign of debate was established as the all-important factor in parliamentary and political life.

"The age of Queen Anne stands out as a distinct epoch in the history of the world. It takes rank with the age of Pericles in Greece, with the Augustan era in Rome, with the Elizabethan era in England. . . ." The greatness of an era is naturally associated with the one whose name is given to it, but in speaking of the age of Queen Anne the author continues: "We cannot possibly associate the greatness of the age with any genius of inspiration coming from the woman whose name it bears. Anne was born to a great era, just as she was born to a crown, and had no more personally to do with the making of its greatness than if she had been born in a garret to a life of commonplace obscurity. Even the worst faults of Elizabeth may be seen to have had some share in creating much of the picturesque greatness at least which belongs to the Elizabethan age. But even the best virtues of Anne had little or nothing to do with the inspiration and promotion of the greatness which marks her reign." During the thirteen years that Anne's brother-in-law, William III., had occupied the throne, he had done much for the country which had elected him as King. "He realised that the days of absolute monarchy were gone by for England. . . . He laid the foundations of the constitutional system by which we mean the political system that depends avowedly on some sort of representative principle, and is not thereby the expression and realisation of the Sovereign's will and pleasure." But William's life was cut short in the midst of a career marked by the single aim of securing for his subjects advantages which would be to their lasting benefit.

His position was a difficult one. Ill-health added to it. The adherents of the Stuarts were in secret opposition As a foreigner he was never particularly popular with the people, and his ways were not the ways of Englishmen. To his wife, Mary Stuart, who was an English princess and Anne's elder sister, he was devotedly attached. When absent from home she had managed affairs with so much wisdom and judgment that everyone was devoted to her. Her death from small-pox at the age of 32 was an abiding sorrow to the stern, silent King. William the Third laid the foundations of the existing order of things into which Anne came when she succeeded him in 1702. He was without doubt "a great statesman as well as a great soldier; he could read the signs of the times. . . . He saw how the new Constitution was to be constructed, and how it was to act in the future. He was like the inventor and constructor of some new model sailing ship, who has got the vessel ready for sea, and has even taken the command, but is for a time prevented by stress of weather from giving his experiment a fair trial, and is cut off by death before the ship can make her first voyage." Anne, who was born to a throne, but was not by temperament a ruler, had the sense to realise the defect, and was singularly fortunate in the statesmen and generals bequeathed to her by the late King. Hers was a nature that required a stronger to lean upon; yet she had much dignity of bearing and "a voice of such sweetness in the pronunciation that it added much life to all she spoke." Upon her accession her reception of the addresses from the two Houses of Parliament impressed the hearers so favourably "that they went from her highly satisfied with her goodness and obliging deportment, for she hearkened with attention to everything that was said to her." The capacity to be, or seem to be, a good listener is an invaluable quality in a new sovereign. She was thirty-eight when she was crowned Queen. On her marriage with Prince George of Denmark, at the age of twenty, Lady Churchill, the beautiful and imperious Sarah Jennings, who became afterwards the Duchess of Marlborough, was appointed a lady of the bedchamber. The Duke of Marlborough had from his earliest years been about the Court, first in the service of Anne's father, James II., and later, after deserting his cause, when he joined William of Orange, and assisted greatly by his extraordinary military and diplomatic gifts in establishing William on the throne of his late master. He was appointed by William governor to Anne's surviving child, the young Duke of Gloucester. "Throughout the whole of her life it was characteristic of Anne to depend upon somebody to take her orders from, and have her path in life, and even her ways of thinking, pointed out to her by them." For many years this occupation was uncompromisingly undertaken by Lady Churchill." Anne's husband can at no time have been her adviser, but with all his faults the Queen was sincerely attached to him; in fact "her affection for him was one of her few strongly developed qualities." She would not entertain the idea of a second marriage. After his death, in 1708, when addresses were presented by the Houses of Parliament requesting her to consider the possibility of one, "the Queen, in graceful, touching, but decisive words, declared that it was impossible for her to act on the recommendation." In politics the names of the Duke of Shrewsbury, the Earl of Oxford, and Viscount Bolingbroke, and in the field those of the Dukes of Marlborough and Ormond. The Earl of Peterborough and others hold a place in history for all time. Anne's reign is made brilliant by the men of letters who distinguished it. Addison, Pope, Swift, were the great writers of her time. In music Handel was the most conspicuous genius. One of the most notable of Anne's good works was the foundation of a charity, still flourishing, as "Queen Anne's Bounty," for the augmentation of the salaries of the poorer clergy. She also restored to the Church much of the property confiscated at the Reformation. Mr. McCarthy concludes his exhaustive study of State and military affairs of the time with this summary: "The reign of Queen Anne must always be regarded as one of the great historical eras forming the landmarks of England's progress in civilisation. . . . Her name is made immortal if only by the mere fact that it was her happy fortune to be England's figurehead at such an epoch. Her name will pass into history with the name of Queen Elizabeth and with the name of Queen Victoria."

* "The Reign of Queen Anne." By Justin McCarthy. (Publishers: Chatto and Windus. 2 Vols. Price 5s.)

### For Reading to the Sick.

"INTO THY HANDS I COMMEND MY SPIRIT."

I wish to have no wishes left
But to leave all to Thee,
And yet I wish that Thou should'st will
Things that I wish to be.

All graces I would crave to have
Calmly absorbed in one—
A perfect sorrow for my sins,
And duties left undone.

I would the light of reason, Lord!
Up to the last might shine,
That my own hands might hold my soul
Until it passed to Thine.

*Faber.*

In the light which streams from the Person and Life of Christ we may see with the eye of faith, that death need not mean the loss, but only the transformation of energy: that what seems outwardly to be an abrupt ending of the activities of the soul as well as of the body, may well be rather the transference of those activities to another sphere of being in the unseen world, to which, if they have come under the wonder-working power of Christ's Life, they have already in their inner reality belonged; so that what in itself, in so far as it belongs to this lower world, is transient and failing and dying away, is but preparing for and leading up to that which is permanent and undying in the world to come.

First that which is natural, afterward that which is spiritual, and the one leading up to the other; but only through suffering, through apparent failure, through death. "That which thou sowest is not quickened except it die." And the suffering, the failure, the death are not merely things to be passed through in order to attain to perfection, but instruments and means towards it. "Our light affliction, which is but for a moment, worketh for us a far more exceeding and eternal weight of glory." St. Paul does not say that it is something to be got over, some obstacle to be overcome before we attain to the glory; but that it works for us the glory—it is the very means of attaining it. All the changes, and trials, and disappointments, and sufferings of this time are the means of perfection, if only we use them rightly according to God's Will, as His children in Christ, partakers of Christ's Life, guided by His Spirit.

*J. W. Hicks.*

Say! say! is it to die—
To give this weary body unto sleeping?
To lay down sorrow's crushing cumbrous load?
To rest where we can hear no sounds of weeping,
Far, far away from life's tear-tracen road?

Oh! this is not to die—
Is it not rather into Life expanding,
Breaking the trial-state to live indeed?
Safe from the tempest in the haven landing,
From storms, from toils, from rocking billows freed?

And yet to earth we die—
Born to new Life with all its weight of blessing,
Born to a world where ills can never press;
Exalted, pure, angelic joys possessing,
If this be Death, then Death is Happiness!

*J. H. Newman.*

### Notes and Queries.

**The Editor is always willing to answer in this column, without any fee, all reasonable questions, as soon as possible.**

**But the following rules must be carefully observed:—**

**1. Every communication must be accompanied by the name and address of the writer.**

**2. The question must always bear upon nursing, directly or indirectly.**

**If an answer is required by letter a fee of half-a-crown must be enclosed with the note containing the inquiry, and we cannot undertake to forward letters addressed to correspondents making inquiries. It is therefore requested that our readers will not enclose either a stamp or a stamped envelope.**

*Maternity.*

(135) I am a monthly nurse, and have practised midwifery for 10 years, and at one time was nurse at the L—— Workhouse Infirmary for 16 months. Will you kindly inform me what steps I should take in order to be registered?—*Nurse Lillie.*

Apply to the Midwives' Institute, 12 Buckingham Street, Strand, W.C.

I am a monthly nurse and hold certificate from a London hospital. Will you tell me where I could obtain my L.O.S. and work in return as payment? I am married, and have to support my home?—*Nurse P.*

Write to the matron of the London hospital where you were trained. Also to the London Obstetrical Society, 20 Hanover Square, London, W., stating what you wish, and ask for syllabus of Examination. This will show you what is required.

*R. N. P. F. N.*

(136) Is there any fund, similar to the Royal National Pension Fund for Nurses, in which nurse-attendants can participate?—*S. W.*

All engaged in nursing can participate in the benefits of the Royal National Pension Fund for Nurses. Write to the Secretary, 28 Finsbury Pavement, E.C., and ask if you are eligible.

*Sanatoria.*

(137) Kindly tell me which open-air sanatorium is the nearest to the West End of London.—*M. B.*

The Mount Vernon Hospital for Consumption, Hampstead, N.W. You can obtain a book giving a list of the principal sanatoria in England from the Manager, the Scientific Press.

*Colosseum Nurses' Association.*

(138) Will you kindly inform me the address of the Colosseum Nurses' Association?—*Co-op.*

In reply to this and several other correspondents, the address is 7 Colosseum Terrace, Regent's Park, N.W.

*Employment for Maternity Nurses.*

(139) I have been trained at Queen Charlotte's Hospital, and hold a certificate for midwifery and the L.O.S. But I cannot find employment, though I have tried every avenue. Can you help me?—*Nurse J.*

There is no agency for monthly nurses, and without general training you cannot join a nurses' co-operation. We can only advise you to watch the advertisement columns.

*Indian Medical Nursing Service.*

(140) Being desirous of becoming a member of the Indian Medical Nursing Service, will you kindly inform me where and how to apply for the necessary forms and rules of admission; and also say if certificates of three years' general training, L.O.S., and massage are sufficient qualifications, or if influence is required.—*M. C. F.*

Do you mean the Indian Nursing Service? If so, apply to the India Office, St. James's Park, S.W. Admission is by merit, not by influence. Your qualifications are good so far as stated.

**Useful Handbooks for Nurses.**

"Nurses' Dictionary of Medical Terms." Cloth, 2s.; leather, 2s. 6d.; post free 2s. 8d.
"On Preparation for Operation in Private Houses." 6d.
"Hospital Sisters and their Duties." 2s. 6d.
"Medical Gymnastics, including the Schott (Nauheim) Movements." 2s. 6d.
"The Human Body." 5s.
"Practical Handbook of Midwifery." 6s.
"A Handbook for Nurses." (Illustrated.) 5s.
"Tendencies to Consumption: How to Counteract Them." 2s. 6d.
"Syllabus of Lectures to Nurses." 1s.

The above works are published by the Scientific Press, Ltd., and may be obtained through any bookseller or direct from the publisher, 28 and 29 Southampton Street, Strand, London, W.C.

## Figures and Tables

**Fig. 2. f1:**
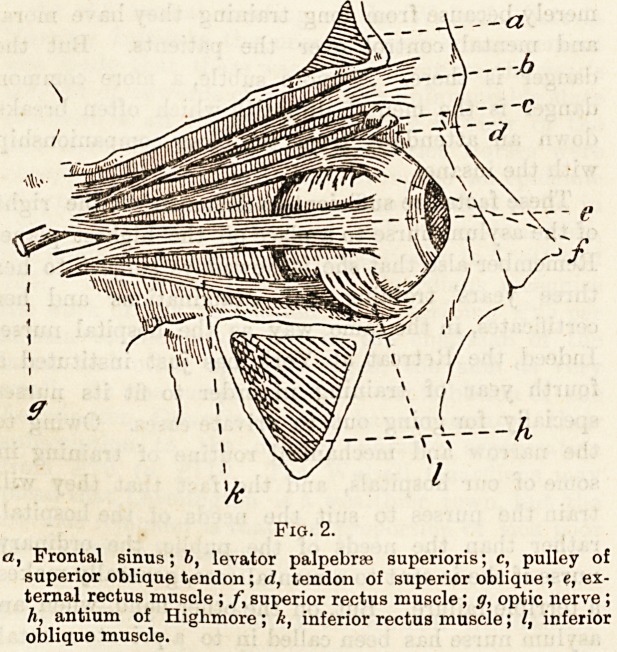


**Fig. 3. f2:**